# Molecular Epidemiologic Evidence for Diabetogenic Effects of Dioxin Exposure in U.S. Air Force Veterans of the Vietnam War

**DOI:** 10.1289/ehp.9262

**Published:** 2006-08-03

**Authors:** Phillip Thomas Fujiyoshi, Joel Edmund Michalek, Fumio Matsumura

**Affiliations:** 1 Department of Environmental Toxicology, University of California-Davis, Davis, California, USA; 2 The University of Texas Health Science Center at San Antonio, San Antonio, Texas, USA

**Keywords:** adipose tissue, Agent Orange, biological markers, diabetes, fasting glucose, glucose transporter type 4, inflammation, molecular epidemiology, NFκB, tetrachlorodibenzodioxin

## Abstract

**Background:**

One of the outcomes positively associated with dioxin exposure in humans is type 2 diabetes.

**Objectives:**

This study was conducted in order to find the molecular biological evidence for the diabetogenic action of dioxin in adipose samples from Vietnam veterans.

**Methods:**

We obtained 313 adipose tissue samples both from Vietnam veterans who were exposed to dioxin (Operation Ranch Hand) and from comparison veterans who served in Southeast Asia with no record of dioxin exposure. We conducted quantitative reverse-transcribed polymerase chain reaction studies on selected marker mRNAs from these samples.

**Results:**

We found the most sensitive and reliable molecular indicator of dioxin-induced diabetes to be the ratio of mRNA of glucose transporter 4 (GLUT4) and nuclear transcription factor kappa B (NFκB), a marker of inflammation. This ratio showed significant correlations to serum dioxin residues and to fasting glucose among those in the Ranch Hand group and, surprisingly, even in the comparison group, who have low levels of dioxin comparable to the general public. Such a correlation in the comparison group was particularly significant among those with known risk factors such as obesity and family history of diabetes.

**Conclusions:**

These results show that the GLUT4:NFκB ratio is a reliable marker for the diabetogenic action of dioxin, particularly at very low exposure levels that are not much higher than those found in the general public, implying a need to address current exposure levels.

Many veterans of the Vietnam War were exposed to 2,3,7,8-tetrachlorodibenzo-*p*-dioxin (TCDD or dioxin), which was present as a contaminant in the herbicide formulation Agent Orange [Booker 2001; Institute of Medicine (IOM) 2000, 2003]. Indeed, significant residues of dioxin have been found in their serum, even after many years, attesting to the fact of their initial exposure and to the long half-life of dioxin, which is estimated to be 7.6 years (95% confidence interval, 7.0–8.2 years) (Michalek and Tripathi 1999). Those dioxin residue levels are relatively high among veterans of Operation Ranch Hand (ORH) (Michalek and Tripathi 1999), the operation responsible for the handling and the actual aerial spraying of Agent Orange and other dioxin-contaminated herbicides in Vietnam from 1962 to 1971. Because of the health implications, many epidemiologic studies have been conducted to identify the possible harmful effects of dioxin on exposed veterans (Frumkin 2003; IOM 2000). Among the recent findings, one of the most consistently identified diseases statistically associated with dioxin exposure is type 2 diabetes mellitus (Henriksen et al.1997; Michalek et al. 1999). This conclusion is also supported by the results of similar epidemiologic studies such as those of the population exposed to high levels of dioxin in Seveso, Italy (Bertazzi et al. 1998; Pesatori et al. 2003), industrial workers (Vena et al. 1998), and Korean Vietnam veterans who were also exposed to Agent Orange (Kim et al. 2003). Despite the epidemiologic evidence for the positive correlation, it has been difficult to explain the mechanistic basis of action of dioxin in causing type 2 diabetes in humans. Because the actual levels of dioxin residues among U.S. veterans are not as high as those of the Seveso population (Bertazzi et al. 1998) or the industrially exposed cohort (Vena et al. 1998) and because small amounts of dioxin and dioxin-type chemicals are also found among general populations in industrialized nations, some scientists question the cause–effect relationship between dioxin residues in humans and an increased incidence of diabetes (Remillard and Bunce 2002).

To address this important environmental health question, we conducted a molecular epidemiologic study on the state of expression of selected biomarkers in adipose tissue samples from participants in the Air Force Health Study. The Air Force Health Study was designed to determine whether veterans of ORH experienced adverse health effects and whether those health effects, if they exist, can be attributed to exposure to herbicides or their dioxin contaminant (Wolfe et al. 1990). The ORH veterans were exposed to herbicides during flight operations, herbicide preparation, and maintenance of the aircraft and herbicide spray equipment. The examination content of the Air Force Health Study emphasized detection of medical end points suspected of being associated with exposure to dioxin, so extensive data on health status and health behaviors were collected on ORH veterans and a matched comparison group of background-exposed veterans. Adipose tissue was selected for study because of its significant role in the etiology of type 2 diabetes (Scheen 2003). Type 1 diabetes, considered genetic with onset at an early age, was not included as an end point in the Air Force Health Study protocol and so was not considered in this study.

We hypothesized two possible pathways for TCDD-induced glucose intolerance. In one pathway involving oncogenes, *Src* [GenBank accession no. M16243 (GenBank 2006)], would activate *MYC* (GenBank accession no. V00568; Jahner and Hunter 1991), which is a direct controller of CCAAT/enhancer binding protein-α (*C/EBP*α; GenBank accession no. NM_004364; Freytag and Geddes 1992). *Src* is known to be essential for TCDD-induced wasting in mice (Dunlap et al. 2002), and *C/EBP*α is a master switch for adipocytes that causes the up-regulation of lipid accumulation processes (MacDougald and Lane 1995). In the other pathway, which involves inflammation, there would be increased production of the cytokine tumor necrosis factor-α (TNF-α; GenBank accession no. NM_000594), one of the major mediators of dioxin-induced cell inflammatory reactions (Alsharif et al. 1994; Kern et al. 2002; Matsumura 2003; Taylor et al. 1992). TNF-α is produced by adipocytes also in response to obesity, causing increased expression of the transcription factor NFκB, and leading eventually to down-regulation of the insulin receptor and the major insulin-responsive glucose transporter GLUT4 (Halle et al. 1998). To test the contributions of each pathway we measured mRNA expression of the genes *c-Src*, *C/EBP*α, *NF*κ*B*, and *GLUT4* in adipose tissue. In addition we measured *GAPDH*, a housekeeping gene (Gorzelniak et al. 2001), for use as a normalization standard to correct for differences in cDNA synthesis efficiency.

## Materials and Methods

The details of basic study design and subject selection have been published previously (Wolfe et al. 1990). The Air Force Health Study compares the health, mortality experience, and reproductive outcomes of ORH veterans with a comparison group of other Air Force veterans who served in Southeast Asia during the same period (1962–1971) the ORH unit was active but who were not involved with spraying herbicides. Comparison veterans were matched to ORH veterans on date of birth, race (black, nonblack), and military occupation (officer pilot, officer navigator, nonflying officer, enlisted flyer, enlisted ground crew). Periodic physical examinations and in-person interviews were conducted in 1982, 1985, 1987, 1992, 1997, and 2002–2003. The methods used in these studies were approved by the institutional review boards at the participating medical treatment facilities. Participation was voluntary, and informed consent was given by subjects at the examination sites.

Dioxin in serum collected from the veterans in 1987 and 1992 was measured by the Centers for Disease Control and Prevention using high-resolution gas chromatography/ high resolution mass spectrometry (Patterson et al. 1987). The between-assay coefficient of variation at three different concentrations of dioxin ranged from 9.4% to 15.5%. For those veterans whose serum dioxin level was below the limit of detection, we assigned a level equal to the detection limit divided by the square root of 2 (Hornung and Reed 1990). Dioxin results were expressed in parts per trillion on a serum lipid weight basis. Fasting glucose (milligrams per deciliter) was determined with Paramax equipment (model 720 ZX; Baxter Scientific Instruments, Deerfield, IL). Body mass index (BMI) was defined as weight divided by the square of height, and percent body fat (PBF) was defined as (1.264 × BMI) − 13.305 (Knapik et al. 1983).

Before the commencement of the 1997 physical examination, 650 veterans were randomly selected according to a four-way factorial defined by service group (ORH, comparison), diabetic status (diabetic, nondiabetic), age (young, born during or after 1942; old, born before 1942), and body fat (lean, PBF ≤ 25; obese, PBF > 25) from those who had been compliant in the 1992 examination (*n* = 2,233) after excluding those who had a missing dioxin measurement (*n* = 78) or diabetes prior to service in Southeast Asia (*n* = 5), died (*n* = 55), or refused to participate (*n* = 6). The desired sample size (*n* = 650) represented 31.12% of the eligible veterans (*n* = 2,089), and a sample including approximately 31.12% was randomly selected from each of the 16 cells. Final numbers are shown in Table 1. Type 2 diabetes mellitus cases were diagnosed during the post-Vietnam period from the end of the veteran’s last tour of duty to 31 December 2000. Veterans who had a verified history of diabetes by medical diagnosis or exhibited a 2-hr postprandial glucose laboratory value of ≥200 mg/dL were classified as diabetic. Veterans not meeting these criteria were defined as nondiabetic.

At the 1997 examination, the selected veterans were invited to submit to an extraction of 2–15 g of lateral abdominal subcutaneous adipose tissue by liposuction. Of these, 107 were excluded because of medications they were taking, 106 were determined to be too lean, 66 refused, 3 were medically deferred, 1 could not be scheduled, and 54 were not asked, leaving 313 to complete the procedure. The specimens were flash frozen in liquid nitrogen at the clinic and shipped to the laboratory in dry ice, thawed on ice, washed in sterile phosphate buffer solution to remove blood, refrozen, and stored at −80°C until sample analysis.

We extracted RNA from approximately 500 mg tissue from each sample using TRIzol LS (Invitrogen, Carlsbad, CA), followed by a digest with RNase-free DNase I (Roche Applied Science, Indianapolis, IN) and a repeat extraction. The yield of RNA was determined spectrophotometrically. Specimens from two individuals were used up in preliminary studies. We synthesized cDNA from 1 μg total RNA using the Omniscript RT Kit (Qiagen, Valencia, CA), modifying the kit instructions by doubling the Oligo-dT primer and 10X buffer amounts and adjusting the water amount to yield 40 μl total volume.

Using conventional *Taq* DNA polymerase (Qiagen), 99 cDNA samples were amplified in duplicate to reduce variation due to saturation effects. Amplified fragments were separated on a 1% agarose gel alongside a DNA ladder of 100-bp increments, which was included to confirm fragment molecular weights. Band density was quantified digitally using the ChemiImager 4400, version 5.5 (Alpha Innotech, San Leandro, CA). Primers used for *GAPDH*, *c-Src*, and *NF*κ*B* were previously described (Ercolani et al. 1988; Kubota et al. 2001; Meyer et al. 1991), whereas we designed primers for *C/EBP*α [5′-TTCCG-GTGCCTCCTGAAAGC-3′ (sense) and 5′-ACAGCCAGATCTCTAGGTCT-3′ (antisense)] and *GLUT4* [5′-CAACTG-GACGAGCAACTTCA-3′ (sense) and 5′-CCAGCTTCCCAATTCTACCA-3′ (antisense)]. Amplification conditions were as follows: 2 min initial denaturation at 94°C, cycling steps of 1 min denaturation at 94°C, 1 min annealing, and 1 min elongation at 72°C, ending with 10 min final elongation at 72°C. Annealing temperatures and cycle numbers were 60°C and 25 for *GAPDH* and *C/EBP*α; 55°C and 25 for *c-Src*; 60°C and 30 for *GLUT4*; and 62°C and 32 for *NF*κ*B*. One sample was designated as the internal standard for polymerase chain reaction (PCR) and repeated in each run and on each gel in order to compare between runs.

The remaining 212 cDNA samples were analyzed without duplication by real-time PCR on the LightCycler (Roche Applied Science) using the QuantiTect SYBR Green PCR kit (Qiagen) (Vogel et al. 2005) for *GAPDH*, *c-Src*, and *GLUT4*, and LightCycler FastStart DNA Master SYBR Green I (Roche Applied Science) for *C/EBP*α and *NF*κ*B*. We designed a new *GAPDH* primer pair using PRIMER 3 (Rozen and Skaletsky 2000): 5′-GAGT-CAACGGATTTGGTCGT-3′ (sense) and 5′-TTGATTTTGGAGGGATCTCG-3′ (antisense). PCR conditions followed kit instructions, with annealing temperatures and extension times of 54°C and 12 sec for *GAPDH*, 53°C and 28 sec for *c-Src*, 55°C and 20 sec for *GLUT4*, 60°C and 28 sec for *C/EBP*α, and 62°C and 17 sec for *NF*κ*B*. We increased the magnesium chloride concentration for the LightCycler kit to 4 mM, and the PCR product size was checked on a 1% agarose gel. We calculated relative sample concentrations from crossing point data using the transform 2^−^*^x^*. To make LightCycler-determined values from different runs comparable to each other and to conventional values, we reran subsets of samples from each run in a common run.

We performed general linear model statistical analysis using SAS software, version 9.1 for Windows (SAS Institute, Cary, NC). We considered and dismissed main effects models of PCR values in terms of dioxin with adjustment for age, body fat, diabetic status, and exposure group because none of these yielded significant results. Subsequently, interaction models involving the product of dioxin with age, body fat, and group were considered; many of these indicated significant interactions, motivating stratification. PCR values were transformed for analysis using various log and power transforms as needed to remove skewness. For purpose of analysis, body fat categories of lean and obese were based on 1997 measurements. All statistical testing was two-sided, with a significance level of *p* = 0.05.

## Results

Raw mRNA values for expression of the four genes studied, before GAPDH normalization, had interquartile ranges of 2.2-fold for *C/EBP*α, 3.3-fold for *NF*κ*B*, 3.4-fold for *GLUT4*, and 3.6-fold for *c-Src*. By comparison, the interquartile range for *GAPDH* was > 6-fold. GAPDH-normalized gene expression values for *c-Src*, *NF*κ*B*, and *C/EBP*α, after transformation, were significantly negatively correlated with transformed *GAPDH* values *p* < 0.001).

**Figure 1:**
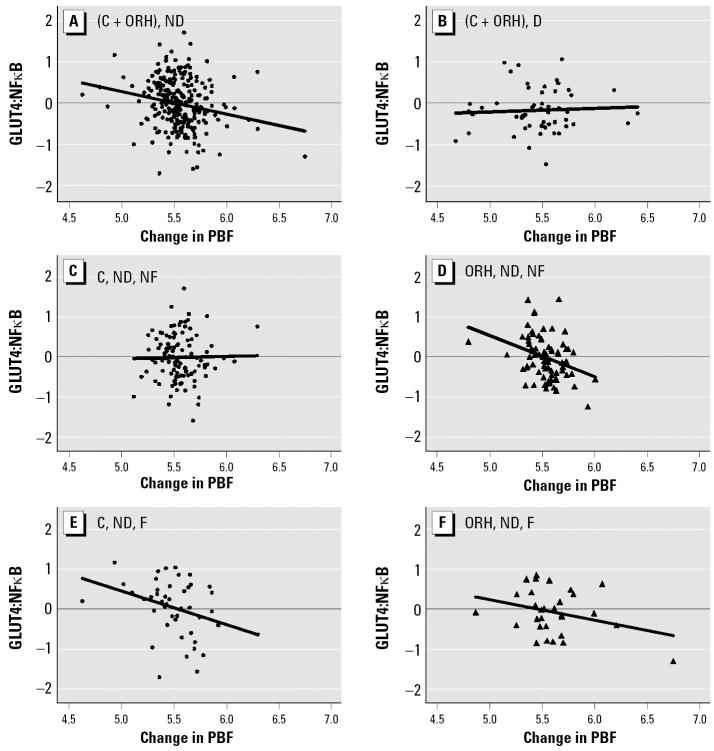
The GLUT4:NFκB ratio versus change in PBF from 1992 to 1997. Abbreviations: C, comparison; D, diabetic; F, family history of diabetes; ND, nondiabetic; NF, no family history of diabetes. Transforms: GLUT4:NFκB, log_10_(*x*); change in PBF, (*x* + 30)^½^. (*A*) Comparison and ORH, ND (*r* = −0.21; *p* < 0.001). (*B*) Comparison and ORH, D (*r* = 0.06; *p* = 0.68). (*C*) Comparison, ND, NF (*r* = 0.04, *p* = 0.7). (*D*) ORH, ND, NF (*r* = −0.34, *p* = 0.003). (*E*) Comparison, ND, F (*r* = −0.33, *p* = 0.03). (*F*) ORH, ND, F (*r* = −0.31, *p* = 0.11). (*A*) and (*B*) slopes are different (*p* = 0.03); (*C*) and (*D*) slopes are different (*p* = 0.02); (*C*) and (*E*) slopes are different (*p* = 0.05); (*F*) slope is not different from (*C*) slope (*p* = 0.16) or (*E*) slope (*p* = 0.51). Correlations are partial; they and interaction *p*-values were adjusted for age.

**Figure 2:**
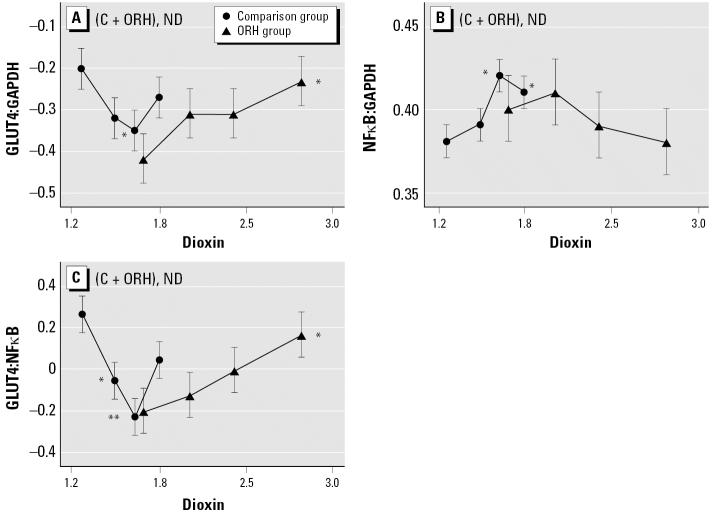
Effect of dioxin load, stratified by quartile, on selected mRNA expression parameters in adipose tissue of nondiabetic subjects by exposure group. Abbreviations: C, comparison; ND, nondiabetic. Transforms: GLUT4:GAPDH, log_10_(0.1 + *x*); NFκB:GAPDH, log_10_(2 + *x*); GLUT4:NFκB, log_10_(*x*); dioxin, 1+ log_10_(*x* − 0.5). (*A*) GLUT4:GAPDH (C trend, *p* = 0.38; ORH trend *p* = 0.05) (*B)* NFκB:GAPDH (C trend, *p* = 0.16; ORH trend, *p* = 0.18. (*C*) GLUT4:NFκB (C trend, *p* = 0.07; ORH trend, *p* = 0.02). Least square means, trend *p*-values, and quartile contrasts were adjusted for age and PBF. *p < 0.05, and **p < 0.01 compared with the mean for the lowest dioxin quartile.

One of the most statistically significant correlations of mRNA expressions or their ratios to change in PBF was the ratio of GLUT4 to NFκB (GLUT4:NFκB ratio) among combined nondiabetic subjects at *p* < 0.001 (Figure 1A). The ratio of GLUT4 to C/EBPα (*p* = 0.002, data not shown) and the ratio of Src to NFκB (*p* = 0.031, data not shown) also showed significant correlations to PBF in the same population. In all cases the value of the ratio tended to drop with increasing PBF among nondiabetic subjects, as shown in Figure 1A compared with the corresponding diabetic subjects, which showed no relationship (e.g., Figure 2B). The differences in these ratios between the non-diabetic and diabetic populations were significant in all three cases. To increase the sensitivity of detection of this physiologic change, we adopted the GLUT4:NFκB ratio as the main marker. Further analyses of nondiabetic subgroups using the same GLUT4:NFκB ratio showed that, among nondiabetic individuals in the comparison group without family history of diabetes, there was actually no negative or positive correlation to body weight change (Figure 1C). In contrast, among the corresponding subgroup (nondiabetic with no family history of diabetes) in the ORH group (Figure 1D), we found a significant negative correlation (*p* = 0.003). Even among nondiabetic comparison subjects, those with a family history of diabetes showed a negative slope (Figure 1E; *p* = 0.03), which was also statistically different (*p* = 0.05) from the slope in Figure 1C.

To study the effect of dioxin on the expression of each marker, we subdivided each group into quartiles according to measured levels of serum dioxin residues and compared gene expression ratios among nondiabetic individuals of these subgroups. GLUT4:GAPDH and NFκB:GAPDH ratios showed significant differences among quartiles (Figure 2A,B). A similar result was obtained when GLUT4 was compared to C/EBPa instead of GAPDH (data not shown). However, the ratio that gave us the clearest trend with dioxin was again GLUT4:NFκB (Figure 2C). The most conspicuous difference between the two service groups was the direction of slopes. Veterans of the first quartile of the comparison group had a significantly higher GLUT4:NFκB ratio than those of the second and third dioxin quartiles, exhibiting a negative relationship to dioxin residues; in contrast, Agent Orange-exposed ORH subjects clearly showed a positive trend. Among comparison subjects, only the highest dioxin residue quartile contradicted the downward trend.

**Figure 3:**
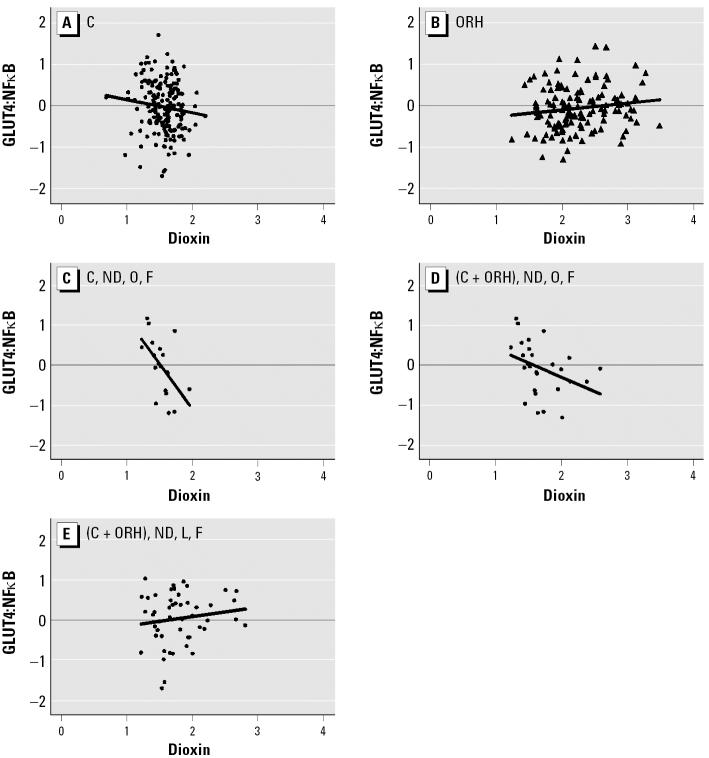
Relationship between serum dioxin levels and GLUT4:NFκB ratio among several subgroups of comparison and ORH populations. Abbreviations: C, comparison; F, family history of diabetes; L, lean; ND, nondiabetic; O, obese. Transforms: GLUT4:NFκB, log_10_(*x*); change in PBF, (*x* + 30)^½^. (*A*) Comparison (*r* = −0.12, *p* = 0.11). (*B*) ORH (*r* = 0.21, *p* = 0.02). (*A*) and (*B*) slopes are different (*p* = 0.01). (*C*) Comparison, ND, obese, F (*r* = −0.58, *p* = 0.02); there is a significant three-way interaction effect of obesity, F, and serum dioxin on GLUT4:NFκB among ND comparison subjects (*p* = 0.045). (*D*) Comparison and ORH, ND, obese, F (*r* = −0.32, *p* = 0.12). (*E*) Comparison and ORH, ND, lean, F (*r* = 0.13, *p* = 0.37). (*D*) and (*E*) slopes are different (*p* = 0.04). All correlations are partial, adjusted for age and PBF.

In view of this finding, we further analyzed the relationships between the GLUT4:NFκB ratio and dioxin exposure in the full cohort of veterans (Figure 3A vs. Figure 3B). In general, dioxin was associated with an increase in the GLUT4:NFκB ratio in the ORH group, whereas there was no trend in the comparison group. Further analyses of several subgroups within each service group revealed that the GLUT4:NFκB ratio tended to decline when serum dioxin increased among nondiabetic individuals in the comparison group who are obese and have a family history of diabetes (Figure 3C). Other subgroups that showed significantly different GLUT4:NFκB responses to increasing serum dioxin were the combined (comparison + ORH) subgroup consisting of obese subjects with a family history of diabetes (Figure 3D) compared with the combined subgroup of lean subjects with a family history of diabetes (Figure 3E).

**Figure 4:**
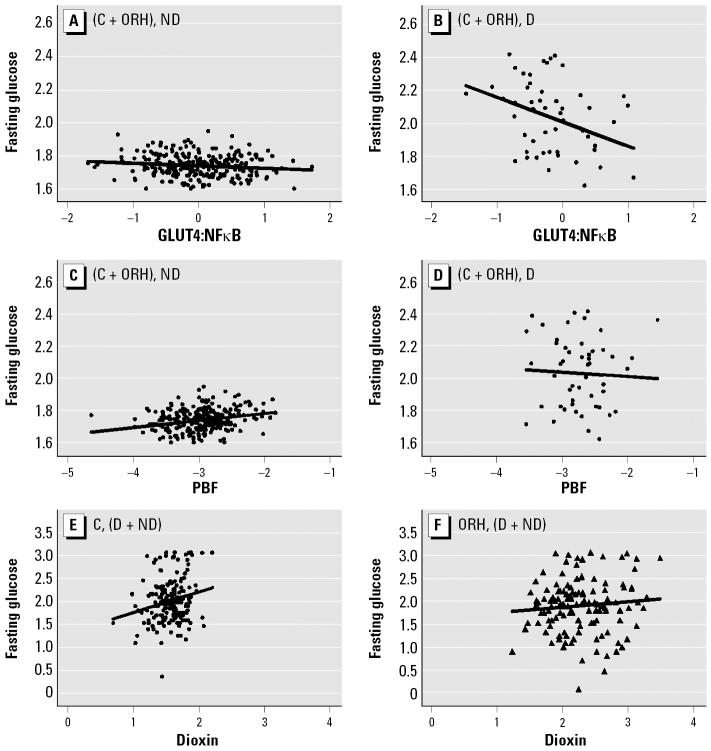
Effects of GLUT4:NFκB ratio (*A, B*), PBF (*C, D*), and serum dioxin (*E, F*) on fasting blood glucose. Abbreviations: C, comparison; D, diabetic; ND, nondiabetic. Transforms: GLUT4:NFκB, log_10_(*x*); dioxin, 1+log_10_(*x* − 0.5); fasting glucose by log_10_(*x −* 40) for (*A–D*) and by 3.1 − 10^7^/*x*^3.5^ for (*E*, *F*) because of the different grouping; PBF by −1/(*x* + 10). (*A*) Comparison and ORH, ND (*r* = −0.10, *p* = 0.13). (*B*) Comparison and ORH, D (*r* = −0.36, *p* = 0.009). (*A*) and (*B*) slopes are different (*p* < 0.001). (*C*) Comparison and ORH, ND (*r* = 0.27, *p* < 0.001) (*D*) Comparison and ORH, D (*r* = −0.05, *p* = 0.73). (*C*) and (*D*) slopes are not different (*p* = 0.09). (*E*) Comparison, D and ND (*r* = 0.17, *p* = 0.02). (*F*) ORH, D and ND (*r* = 0.07, *p* = 0.40). (*E*) and (*F*) slopes are not different (*p* = 0.19). Correlations are partial; they and interaction *p*-values were adjusted for age and PBF where appropriate.

To test the validity of using the GLUT4:NFκB ratio as a molecular parameter to assess diabetogenic conditions, we analyzed its relationship to the high levels of serum glucose after fasting (fasting glucose). As seen in Figure 4A, the fasting glucose levels stayed within a narrow range of values among all (comparison + ORH) nondiabetic study subjects over the wide range of GLUT4:NFκB ratios, as expected. In contrast, among all diabetic subjects (Figure 4B), those with lower GLUT4:NFκB ratios exhibited higher fasting glucose levels. We further checked the relationship between PBF and fasting glucose levels in all nondiabetic subjects (Figure 4C) compared with all diabetic subjects (Figure 4D). We found a significant relationship in non-diabetic subjects (*r* = 0.27, *p* < 0.001) but not in diabetic subjects (*r* = −0.05, *p* = 0.73).

Next, we studied the relationship between the levels of fasting glucose and serum dioxin to further test our premise that dioxin acts as a diabetogenic agent. When both nondiabetic and diabetic subjects were combined within each service group, we found a positive correlation between fasting glucose and serum dioxin levels in the comparison group (Figure 4E; *p* = 0.02). In contrast, these two parameters were not correlated in the corresponding ORH subgroup (Figure 4F).

## Discussion

Initially we conducted a preliminary survey of the expressions of the proposed markers in all samples to see which markers or their combinations would give a reliable indication of physiologic conditions leading to diabetes. To aid in this process, we formulated a working hypothesis that the diabetogenic action of TCDD could be phenotypically similar to that of obesity. Therefore, we studied the relationship between mRNA expression of those selected markers and PBF gain over the last 5 years among veterans. Of all of the molecular markers and their combinations examined, the most readily recognizable gene expression effects were those of the GLUT4:NFκB ratio in response to the presence of dioxin, as assessed in the serum of the veterans. This observation agrees well with the generally accepted view that in the case of obesity-induced type 2 diabetes TNF-α plays the central role through its action to activate NFκB, which down-regulates *GLUT4* (Ruan et al. 2002). Theoretically, if the *TNF-*α–*NF*κ*B* pathway is activated, a rise in *NF*κ*B* expression will lead to a drop in *GLUT4* expression; thus, the ratio is expected to be more responsive than either individual gene normalized by a housekeeping gene. The trends for GLUT4:C/EBPα and Src:NFκB may represent merely weaker responses of ratios that contain the GLUT4 or NFκB component of the GLUT4:NFκB ratio.

This GLUT4:NFκB ratio marker approach was most effective in detecting the effect of dioxin at low-to-medium levels of exposure, corresponding to the lower three quartiles in the comparison group (Figure 1). This range of dioxin levels corresponds to 1–5.3 ppt. In fact the highest dioxin level for the entire comparison group is 16.3 ppt. The level of dioxin in serum lipids among U.S. workers that Sweeney et al. (1997–1998) found in their comparison group was 0–20 ppt, indicating that the range we found in the comparison group in the present study was not much different from that of the general public in the United States. In this regard, it is important to point out that we found significant signs of dioxin-correlated diabetogenic tendency among comparison group subjects with low levels of dioxin, particularly in those with genetic (family history of diabetes) and physiologic (obesity) risk factors (Figure 3C). Furthermore, dioxin, even at this low concentration range, definitely has an effect on the levels of fasting glucose (Figure 4E). The GLUT4:NFκB response to dioxin exposure levels found in the lower three quartiles of the comparison group also agrees well with the data obtained from cell models (Ruan et al. 2002) and animal models (Dunlap et al. 2002; Liu and Matsumura 1995).

The observation that the GLUT4:NFκB ratio declined with weight gain, independent of exposure history (Figure 1A) and with higher serum dioxin residues among comparison individuals (Figure 2C), particularly obese individuals with family history of diabetes (Figure 3C), suggests that dioxin works synergistically with known diabetes risk factors to alter glucose metabolism in a way that resembles the inflammation mechanism of weight gain. The GLUT4:NFκB ratio appears to be a useful biomarker for the detection of the diabetogenic action of these factors. The inflammation mechanism also seems to operate at low background levels of dioxin, as seen in the three lowest serum dioxin quartiles (i.e., those with ≤ 5.3 ppt) of the larger group of background-exposed veterans (Figure 2C). Such an observation alone would not constitute a proof for the identical action mechanism of dioxin to diabetogenic action of obesity, but it allows us to formulate a hypothesis along this line of logic to help our future studies.

We found the GLUT4:NFκB ratio to be a reliable parameter in assessing the state of diabetes. Diabetic subjects with lowered GLUT4:NFκB were less able to regulate blood glucose, whereas nondiabetic subjects maintained fasting glucose levels within a narrow range of values, independent of GLUT4:NFκB (Figure 4A,B). However, our finding regarding this effect of GLUT4:NFκB was somewhat surprising in view of the lack of correlation of GLUT4:NFκB with weight gain among diabetic subjects (Figure 1B); this finding suggests that once the study subjects develop diabetes, the GLUT4:NFκB ratio does not work well as a biomarker for obesity. However, the GLUT4:NFκB ratio does work as a biomarker for elevated fasting glucose (Figure 4B). The important message derived from Figure 4B is that the workable range or the usefulness of the GLUT4:NFκB ratio as a biomarker depends largely on the factor against which it is being regressed. Thus, one should not assume automatically that the state of disease makes it impossible to use this biomarker on all diabetes-related cellular changes.

Among nondiabetic veterans without a family history of diabetes (i.e., healthy sub-population) in the comparison group, we found no detectable effect of obesity on the GLUT4:NFκB ratio (Figure 1C), indicating the existence of normal homeostatic control. In contrast, the corresponding subgroup from the ORH group clearly showed the effect of obesity (Figure 1D) as though they already had the genetic risk factor, as in the case of the subgroup with a family history of diabetes in the comparison group (Figure 1E). This set of data also supports our interpretation that dioxin acts as one of the risk factors for diabetes.

As we expected, fasting glucose levels were reliable parameters in judging the diabetogenic effects of obesity among nondiabetic subjects (Figure 4C) at the early stage of developing diabetes but not at the later stage, as seen in diabetic subjects (Figure 4D). In addition, the observation that fasting glucose is higher among subjects in the comparison group who had higher serum dioxin levels complements the observation of Henriksen et al. (1997), who found increased fasting glucose with higher levels of dioxin in a larger cohort of Agent Orange–exposed veterans.

The decrease in the expression of *GLUT4* in adipose tissue has been shown to be associated with non–insulin-dependent diabetes. Minokoshi et al. (2003) found that tissue-specific ablation of *GLUT4* and insulin receptor in adipose tissue or muscle in mice led to insulin resistance and diabetes in the mice lacking adipose *GLUT4* expression but not in those missing *GLUT4* only in muscles. In humans, Garvey et al. (1991) found that an 80% decrease in GLUT4 protein per cell in the adipocytes of diabetic subjects compared with lean nondiabetic controls was associated with a 56% decrease in maximally insulin-stimulated glucose uptake. The level of GLUT4 mRNA was correlated with the amount of GLUT4 protein (*r* = 0.77) in their controls but not in their diabetic subjects.

There are also precedents indicating that the change in NFκB is correlated to diabetes. NFκB is a nuclear transcription factor that is known to be activated by inflammatory signaling of several agents, including TNF-α, and to transmit their messages into the nucleus. Although we did not include TNF-α in the present study, its involvement in the toxic action of dioxin is well known (Alsharif et al. 1994; Taylor et al. 1992). TNF-α is one of the major mediators of dioxin-induced cell inflammatory reactions (Matsumura 2003). Furthermore, the role of TNF-α in the development of insulin resistance and type 2 diabetes, particularly in the case of obesity-induced diabetes, is now becoming well recognized (Das 1999). Indeed, obesity induces increased expression of *TNF*-α and *NF*κ*B*, leading to down-regulation of insulin receptor and decreasing expression of *GLUT4* (Halle et al. 1998). Our observation in the present study that the GLUT4:NFκB ratio dramatically decreases among nondiabetic veterans who experienced a relatively recent increase in body fat attests to the correctness of this diagnosis.

We did not include measurements of other compounds with dioxin-like activity because these were not available in this population until well after the period of this study. Sweeney et al. (1997–1998) found that use of toxic equivalents (TEQ; the body burden of TCDD-equivalent activity from all compounds) led to a narrowing of the differences between acute- and background-exposed populations. Furthermore, the phenoxy herbicides in the Agent Orange formulation are known peroxisome proliferators and thus may have antidiabetic action (Remillard and Bunce 2002). With such confounding factors acting to obscure the effects of TCDD, the results we did find are all the more noteworthy.

One major question that remains unanswered is why the overall relationship between the GLUT4:NFκB ratio and serum dioxin levels show the “V” shape (Figure 2C), indicating a reversal of dioxin’s effect at levels higher than the background range. This is puzzling because, in all other cases, either the GLUT4:NFκB ratio or dioxin levels showed straightforward relationships to other parameters analyzed. One possibility is that veterans with dioxin residue levels > 5.3 ppt are experiencing the effects of cachexia, a typical effect of dioxin exposure. It involves massive loss of adipose tissues in most animals studied, including humans (Matsumura 2003). In this regard, it is interesting to note the similarities in the direction of slopes between Figure 3A and Figure 3D, and between Figure 3B and Figure 3E, indicating that as a whole, comparison subjects are similar to combined (comparison + ORH) nondiabetic obese subjects with a family history of diabetes with respect to their GLUT4:NFκB response to dioxin. In the same manner, ORH subjects are similar to combined (comparison + ORH) nondiabetic lean subjects with family history of diabetes, despite the fact that there is no difference in the frequency of obesity between comparison and ORH subjects. Such an observation favors the view that wasting syndrome is already taking place among those exposed to high levels of dioxin and that ORH subjects as a whole are responding to dioxin as though they were lean. Another possibility is that in human adipose tissues, unlike the case of mice, chronic exposure to high concentrations of dioxin could trigger a strong negative feedback reaction through activation of major “negative regulators” to counteract excessive inflammatory insults. The observation that three of the parameters we studied, *NF*κ*B*, *C/EBP*α, and *GLUT4* expression, show this phenomenon of “reversal” at high dioxin concentrations > 5.3 ppt supports this interpretation. Nevertheless, much more work is needed to prove or disprove these hypotheses. It is also important to point out that, despite the above “reversal” phenomenon in the relationship between GLUT4:NFκB ratio and dioxin, all non-diabetic individuals with significant dioxin residues, including those with residue levels > 5.3 ppt, still show the clear sign of the obesity-related risk of diabetes, judging by the results of experiments shown in Figure 4C.

## Conclusions

In conclusion, by using this molecular epidemiologic approach we found definitive evidence indicating that a diabetogenic shift occurred in the biochemistry of adipose tissues from Vietnam veterans who were exposed to dioxin-containing Agent Orange herbicide preparations. Such a diabetogenic effect of dioxin was observed even among comparison group veterans, particularly those with diabetes risk factors such as obesity and/or a family history of diabetes, despite the fact that their levels of exposure are not really different from those of the general public in the United States. The major implication of the present study is, therefore, that the potential health hazard of dioxin and active dioxin-type chemicals, even at the current level of public exposure, must be taken seriously. Further research is needed to fully elucidate the precise mechanism through which dioxin promotes type 2 diabetes in humans.

## Figures and Tables

**Table 1:** Sample sizes and demographics.

Group/diabetic status	Age category	Lean (*n*)	Obese (*n*)	Total (*n*)
Comparison				
Nondiabetic	Young	58	18	76
	Old	56	24	80
Diabetic	Young	3	7	10
	Old	9	9	18
ORH				
Nondiabetic	Young	46	13	59
	Old	40	11	51
Diabetic	Young	4	4	8
	Old	6	5	11
Total				313
